# *Walking a tightrope* – as a next-of-kin to an adolescent or young adult with cancer facing eating difficulties

**DOI:** 10.1080/17482631.2022.2121029

**Published:** 2022-09-08

**Authors:** Marie Ernst Christensen, Anita Haahr, Pia Riis Olsen, Hanne Krogh Rose, Annelise Norlyk

**Affiliations:** a Research Unit for Nursing and Health Care, Department of Public Health, Aarhus University, Aarhus, Denmark; b Research Centre for Health and Welfare Technology, Program for Rehabilitation, via University College, Aarhus, Denmark; c Department of Oncology, Aarhus University Hospital, Aarhus, Denmark; d Department of Health and Nursing Science, Faculty of Health and Sport Sciences, University of Agder, Grimstad, Norway

**Keywords:** Adolescents/Young adults, cancer, eating difficulties, family/next-of-kin support, high-emetogenic chemotherapy, lack of appetite, meals, nausea

## Abstract

**Purpose:**

Eating difficulties cause reduced food intake and poor quality of life among adolescents and young adults (AYAs) with cancer. Therefore, next-of-kin eating support is crucial. The purpose of this study was to explore the lived experiences of being close to AYAs with cancer in the context of eating when they are at home between high-emetogenic chemotherapy (HEC) sessions.

**Method:**

In-depth interviews were conducted with 12 next-of-kin to AYAs (15–29 years old) with oncological or haematological diseases, treated with HEC. Van Manen’s hermeneutic-phenomenological approach guided the design.

**Results:**

The essential meaning of the next-of-kin experiences is reflected in the overarching theme *“Utilizing meals as an action-opportunity”* consisting of two subthemes: *’Being on constant alert’ and “Walking a tightrope to maintain usual everyday life.”*

**Conclusions:**

Findings revealed that utilizing meals as an action-opportunity towards AYAs’ food intake involved existential feelings including fear of losing their loved ones. Next-of-kin experienced that providing support through and with food was their only avenue of action. However, this sparked feelings of frustration and powerlessness.

## Introduction

Eating is a necessity of life, often taken for granted and performed automatically. However, following high-emetogenic chemotherapy (HEC), eating is a complicated process that places great demands on patients with cancer and their next-of-kin. This study focused on adolescents and young adults (AYAs) aged 15–29 years with cancer and their next-of-kin (Regeringen [Danish Government], 2016).

Eating difficulties may for instance, be caused by taste changes, poor appetite, nausea, and vomiting. These symptoms are closely related to reduced food intake and poor quality of life and are frequent in patients receiving HEC (Brinksma et al., 2015, 2020; Drareni et al., 2021; Loeffen et al., 2015). Nausea and vomiting, in particular, are more prevalent among AYAs than among younger or older patients (Beauchemin et al., 2020; Brinksma et al., 2015, 2020; Linder et al., 2017; Loeffen et al., 2015). Thus, eating difficulties may cause malnutrition in this group (Beauchemin et al., 2020; Brinksma et al., 2015, 2020; Linder et al., 2017; Loeffen et al., 2015). Among AYAs with cancer, the prevalence of malnutrition has been reported to be 75% (Joffe & Ladas, 2020), leaving AYAs at considerable risk of poor clinical outcomes, increased toxicity to chemotherapy and increased susceptibility to infections and morbidity if their eating difficulties are not properly managed (Arends et al., 2017; Joffe & Ladas, 2020). Furthermore, AYAs have a higher mortality and lower five-year survival than younger children, and the prognosis for AYAs with cancer has not been improved as much as the cancer prognosis for children and adults (Bleyer et al., 2017; Nass et al., 2015).

Living with cancer and receiving invasive treatment can be exhausting and may destroy the usual pleasure of eating (Christensen et al., 2021). Mealtimes provide much more than just nutrient supply as they may bring socializing, pleasure and enjoyment, thus affecting the AYA’s entire lifeworld. For example, Guzik et al. (2020) found that AYAs experienced a loss of control over their lives due to their cancer diagnosis and that they felt that cancer influenced their lives, appetite and what they were able to do in their everyday lives. This challenges the provision of eating support among this population even more. In the adult cancer population, Koshimoto et al. (2019) found that outpatients receiving chemotherapy often encountered conflicts over food such as disagreements what is served or how much is consumed with people around them and that mealtimes often led to conflicts with family members. Thus, eating is reported to be difficult not only for the AYAs receiving HEC, but also for supporting family members (Decker, 2007; Stenberg et al., 2012; Williams & McCarthy, 2015).

Paediatric oncology literature recognizes that childhood cancer requires a comprehensive and interdisciplinary care from diagnosis to end of treatment, and that family support plays an important role in overcoming challenges throughout the trajectory of cancer (Kazak, 2006; Treadgold & Kuperberg, 2010). Parents and next-of-kin play a valuable supporting role by accompanying the child daily and assuming responsibility for its care and development (Long & Marsland, 2011). This is also the case for AYAs receiving social support from family or healthcare professionals who positively influence their coping and rehabilitation process (Decker, 2007; McNeil et al., 2019; Pennant et al., 2019). In a recent study, AYAs described close family members, i.e., mothers, fathers, sisters, brothers, spouses, and partners, as their primary sources of social support and highlighted this as the most helpful aspect experienced during their cancer course (Pennant et al., 2019). Especially assisting with everyday tasks at home including providing food was of high importance (Pennant et al., 2019). However, supporting a close relative through cancer comes at a cost. Several studies have examined how providers of social support experience extreme distress when standing by a loved one who is receiving chemotherapy (Bell, 2009; Deshields et al., 2012; Hofman et al., 2004; Matthews et al., 2003; Ream et al., 2021). As chemotherapy now typically is provided as outpatient treatment, the risk of fear and anxiety among support givers are exacerbating and they often feel left alone (Deshields et al., 2012; Matthews et al., 2003; Ream et al., 2021). Consequently, the home context becomes highly relevant as patients’ lives unfold here between chemotherapy treatment series, and next-of-kin are needed to manage the multifaceted chemotherapy-induced side-effects (Deshields et al., 2012; Matthews et al., 2003; Ream et al., 2021).

However, little is known about how cancer and its treatment influence eating and how eating support unfolds between the AYAs and their next-of-kin outside the hospital setting. In addition, the actual experience of the next-of-kin providing eating support for their AYA remains under-examined. Therefore, the purpose of this study was to provide an in-depth understanding of next-of-kin’s lived experiences of being close to an AYA with cancer in regard to eating when residing in their home between HEC sessions. Such knowledge has the potential to heighten our understanding of next-of-kin eating support, develop the health professional´s guidance and therefore improve AYAs’ eating abilities, increase their food intake and ultimately enhance their chances of recovering.

## Methods

This was a hermeneutic-phenomenological study (Van Manen, 2014) designed to capture the lived experiences of being a next-of-kin to an AYA with cancer receiving chemotherapy.

### Participants

Participants were recruited in three oncology departments at a Danish university hospital between August 2019 and May 2020. People were eligible for participation if they were next-of-kin to an AYA aged 15–29 years with oncological and haematological diseases who was being treated with HEC. Next-of-kin to AYAs (five females and seven males aged from 17 to 29 years who were undergoing active HEC) were included in the study. Next-of-kin was defined as people with a close relation, i.e., mothers, fathers, sisters, spouses, or partners to the AYA, who supported them through their cancer course. The sample comprised 12 next-of-kin; one father, two partners/spouses, two sisters, and seven mothers. Additional demographic characteristics are omitted due the phenomenological origin of this study (Norlyk & Harder, 2010).

### Data collection

Data were collected through in-depth, audiotaped interviews. Half of the interviews were held as face-to-face interviews in the homes of the next-of-kin. The remaining interviews were held as telephone interviews. Due to the Covid-19 pandemic, participants were contacted either by telephone or by text message, depending on their preferences to arrange a suitable time for the interview to take place.

Each interview focused specifically on capturing the lived experiences of being a next-of-kin to an AYA with cancer in regards to eating when they were at home between chemotherapy sessions. According to van Manen (Van Manen, 2014), the five existentials; lived body, lived relation, lived space, lived time, and lived things permeate the lifeworlds of human beings and may serve as key terms in interviews using a phenomenological approach (Van Manen, 2014). The opening question was “Please tell me what it is like to be close to an AYA with cancer”. Hereafter, participants were encouraged to elaborate on their experiences of supporting AYAs’ eating through prompts such as; “Please try to tell me about a special moment when … ” or “Please try to give me concrete examples of … ”. The interviewer strived to establish an inviting and open context through active listening, thereby encouraging the next-of-kin to provide concrete descriptions of their lived experiences.

### Data analysis

The interviews varied in duration from 29 to 67 minutes, they were transcribed verbatim, and the data analysis followed the analytical approach described by van Manen (Van Manen, 2014). In the holistic approach, transcripts were read repeatedly keeping an open-mind towards the lived experience descriptions in order to capture an initial sense of “what was going on” (Manen van, 1997; Van Manen, 2012). The interviews were attended in full in an attempt to grasp the main significance of the text. Thereafter, one phrase expressing the essential meaning was composed, such as; *It is fundamental to provide food care to the AYAs despite any conflicts and consequences this may entail*. Furthermore, in the selective approach, the text was condensed into clusters of meanings by searching for the experience of being a next-of-kin in relation to eating within and across interviews. By continuously moving back and forth in an iterative process between clusters and the full interview text, each meaning cluster was analysed and interpreted in the context of the overall understanding of the interviews. Finally, in the detailed approach, meaning clusters and selected meaning units were aggregated into essential themes to capture the phenomenon of being a next-of-kin to an AYA with cancer in relation to eating (Van Manen, 2012). The process of analysis was discussed repeatedly among authors (Manen van, 1997). To enhance the meaning of the experiences described, the meaning units are presented as quotes.

### Ethical considerations

Participants were provided with oral and written information before inclusion in accordance with the Helsinki Declaration (General Assembly of the World Medical Association, 2014). Additionally, they signed a statement of consent, thereby acknowledging that they were aware that participation was voluntary and that they could withdraw at any time during the study course. The Danish Data Protection Agency approved the study (record number 016–051-000001, 144).

## Findings

### Utilizing meals as an action-opportunity

Utilizing meals as action-opportunity was the overarching theme covering the next-of-kins’ experiences of being a close relative to an AYA with cancer in relation to eating. The next-of-kin experienced eating as a complex and highly emotional situation that evoked existential feelings of fear of losing their loved ones and made them eager to save their loved ones from dying of cancer. They experienced being in a difficult supporting position where the use of meals to take action became essential to support the AYA in the best possible way through this tough time in life. Utilizing meals as an action-opportunity embraces this complexity by enhancing that meals are essential for every human being and that “taking care of meals” allowed the next-of-kin to “take action” throughout the cancer trajectory. Thus, meals became a symbol for taking action against the eating difficulties faced by the AYAs. Next-of-kin experienced a basic feeling of responsibility for supporting their loved ones through their illness but needed to strike a balance between their strong sense of duty on one hand and the need to maintain normal everyday routines on the other. The essential meaning of *“Utilizing meals as an action-opportunity”* is further unfolded through the themes *´Being on constant alert´* and *´Walking a tightrope to maintain usual everyday life´*. These themes covered variations of the lived experiences of being a next-of-kin to an AYA with cancer in regards to eating.

### Being on constant alert

The next-of-kin experienced being on constant alert in their efforts to support the AYA’s eating abilities. They naturally accepted the responsibility for providing care by offering their support and did their best to increase the AYA’s food intake. The next-of-kin developed a strong willingness to do whatever they were capable of to make the course of treatment as good and painless as possible. These feelings of responsibility activated an urge to take action and support the AYA’s food intake. Specifically, their urge to take action triggered a primordial force in the next-of-kin and could produce a desire to employ force-feeding. *“I almost wanted to stuff the food into his throat, but I just had to accept that he is a grown-up and he knows what he wants. It does not help to push him”* (Mother to a 22-year-old male).

In addition, the next-of-kin experienced an intense sense of responsibility in their collaboration with the healthcare professionals’ treatment at the hospital, which they made considerable efforts to underpin. They experienced taking responsibility that the AYAs maintained their weight and would occasionally state that they had failed if the AYA had lost weight. This could lead to feelings that they were betraying their role as next-of-kin. *“I was actually horrified that he was losing so much weight. Oh boy. I thought: ‘Why haven’t I been more aware of it?’ It was because the diagnosis took up a lot more space and I was unaware of his eating pattern. It was just not the highest priority right then and there. However, it became a priority!”* (Mother to a 22-year-old male).

The next-of-kin experienced an intense desire to support the AYAs even though they felt uncertain about what constitutes good and appropriate eating support. Being on constant alert was triggered by information from the hospital explaining that AYAs should eat whatever and whenever they preferred, which the next-of-kin perceived as a carte blanche to eating fast food any time. The next-of-kin would experience a sense of mistrust towards the authorities and felt that they had to compromise on their own convictions about the importance of healthy homemade food when undergoing chemotherapy treatment. On one hand, they wanted to meet the AYA’s wishes and on the other, this challenged their convictions and usual food standards of not having fast food every day, thus triggering a constant state of alertness. They tried to help AYAs control their intake of fast food and attempted fully to understand their urges and desires. *“He must have something to eat, if he is able to eat. He may feel an urge for Mc. D. or pizza even without feeling unwell and then we do not always comply with his wishes. Otherwise, junk food can easily get out of hand”* (Mother to a 17-year-old male).

If the AYAs felt a reduced appetite, this triggered an inner struggle in the next-of-kin that forced them to take action and search for acceptable solutions. They compromised on their usual eating standards as they seemed challenged by the AYAs’ limited eating possibilities. Eating in the sofa was, for example, not in line with the normal routine of having meals while gathered around the dinner table. However, if this would increase the AYAs’ food intake, it was accepted as a new and cozier eating space.

#### Walking a tightrope to maintain usual everyday life

The next-of-kin experienced constantly walking a tightrope to maintain usual everyday life. They needed to strike a balance between their own, the AYA’s, and the other close family members’ needs and strove to stick to normality when everything around them seemed chaotic. They experienced having to act like the family’s coordinator by showing care and consideration for the AYA, but also for everyone else in the family. This included a need to sustain the family’s pre-existing eating standards, eating patterns, eating traditions, and eating times. For example, the next-of-kin strove to preserve mealtimes as the main gathering point by taking into consideration everyone’s needs and wishes. *“If he is unwell, I sit next to him tousling him on his back. However, I also take part in the meals with the others. Of course, I ask if he wants to join. Sometimes he sits with us and then he walks away again”* (Mother to a 17-year-old male).

Next-of-kin experienced walking a tightrope when supporting AYAs’ eating abilities. They were clear about the fact that providing support through and with food was the only thing that they were capable of doing. Knowing that their AYAs would feel better if they ate, but not being able to force their intake of food would create feelings of frustration and powerlessness. It was difficult for them to stand so close to the AYAs being unable to ensure that they had an adequate food intake and this triggered a desire for doing whatever possible, i.e., a wife added more cream to the sauce; a sister was constantly pampering while performing eating support, and mothers provided care in all imaginable situations. *“It was my job to get him to eat. That is what I have been thinking. He is my boy; therefore, it is my job to get him through this”* (Mother to a 22-year-old male). However, the next-of-kin needed to strike a balance between placing the AYA under pressure to improve their food intake and not pressing them excessively. They struggled to find confidence that the AYAs would ask for eating support themselves if they needed it. *“I can´t get him lifted out of his shell again. What can I do for you? Help me. What do you want? Do you have an idea yourself? I will drive you to the end of the world—as long as you get what you want to eat”* (Father to a 19-year-old male).

A risk was present of enmity and conflicts between next-of-kin and AYAs when AYAs felt that there was too much talk about food or believed that they were being pushed too hard to eat. The next-of-kin had to possess a certain amount of indulgence and flexibility. They experienced being scolded, and to avert escalation of conflicts they tried to back off and limit the AYA’s frustrations. However, the next-of-kin could experience the AYAs as unreasonable when they reprimanded them and wanted to take control in the kitchen. Thus, their mutual relationship of trust was being put to the test. The next-of-kin experienced criticism being directed towards them if the AYA, e.g., suspected that strict hygiene rules had not been met or mistrusted the manner in which meals had been prepared. This could create a tense atmosphere around the table, which affected every member of the family, and the next-of-kin would then feel responsible and experience a need to patch things up. *“She does not have to keep an eye on me. She does not have to be a police officer. She just has to trust me, but it is a mother’s job to show patience and forbearance”* (Mother to a 27-year-old female).

## Discussion

The present study illuminates how AYAs’ cancer disease affected the entire lifeworld of their next-of-kin by creating complex and highly emotional situations, which evoked existential feelings in the next-of-kin of fear of losing their loved ones. The next-of-kin experienced meals as an opportunity allowing them to take action and to contribute, which provided the promise of actively letting them participate in the course of AYA’s cancer treatment to save their loved ones from dying. Our findings show that the next-of-kin inevitably took on a supporting role by assuming responsibility, providing assistance and accepting their duty to assist the AYAs in countering their eating difficulties. Furthermore, our findings illustrate that providing care for your child or close relative manifested itself as an absolute basic and fundamental task. This may be viewed through the lens of van Manen’s parenting studies and his conceptualization of lifeworld existentials (Van Manen, 2014). Using the concept of the lived body, Van Manen (2012, 2014) demonstrated that parents experienced their children as utterly separated from them, even though they were physically close. He argues that, for many people, a deep significance lies in the knowledge that parents and children are of one flesh (Van Manen, 2012, p. 105). Thus, in our study, next-of-kin embody their parenthood by looking with parenting eyes at the AYA, for example, lying nauseous and unable to eat in his or her sickbed. This unconditional way of “seeing” is prompting them to take action the way a parent or next-of-kin is supposed to act. The lived body in this study influenced the other dimensions of the lifeworld, such as lived space and lived relation. The home of the parents or next-of-kin is the location of a shared lived space. Here, the AYAs are offered the opportunity to endure their cancer illness and chemotherapy within a safe haven. The experience of home as space may turn out to be supportive or neglectful, open or smothering, liberating or oppressive for the AYA (Van Manen, 2014). The next-of-kin/AYA relation is experienced as a special lived relation to the other. The relation is highly personal and carries interpersonal significance. In our study, the next-of-kin experienced a fundamental need for rendering help and eating support for the AYAs. However, our findings also revealed that their relation was put to the test when the next-of-kin was more demanding than the AYAs found suitable, which would affect the AYAs’ willingness or ability to eat. Conversely, several studies have reported that families were the most important source of support and parental assistance in, e.g., cooking and eating, and that this was highly appreciated among AYAs (Ang et al., 2018; McNeil et al., 2019; Pennant et al., 2019; Wicks & Mitchell, 2010).

The present study contributed to existing knowledge by illuminating that the next-of-kin experienced being on a constant alert with a prominent and strong willingness to do whatever they were capable of doing. Thus, the next-of-kin experienced a strong feeling of responsibility, which activated an urge to take action and support the AYAs’ food intake. Furthermore, they faced the fact that supporting through and with food was the only avenue of action they could take. With reference to the philosopher Levinas, van Manen points out that “care *is* worry” (Van Manen, 2002, p. 270). Van Manen further adds that the more one cares for a person, the more one worries, and the more worries, the stronger is the desire to care (Van Manen, 2002, p. 270). Thus, one may argue that the exorbitant desire to care found in the present study may potentially cause inconvenient conflicts between next-of-kin and AYAs resulting in unsuccessful attempts to enhance food intake. Our findings showed that next-of-kin experienced walking a tightrope as they needed to put pressure on their AYA without pressing excessively. Consequently, if AYAs felt that they were experiencing too much talk about food or were being pushed too hard towards eating; this could cause animosity and trigger conflicts between the next-of-kin and the AYAs. These findings are in line with those of Hopkinson (2018) and Reblin et al. (2019) who found that meals could contributory factor to conflict. However, as described by Reblin et al. (2019), additional conflicts may occur in the young adult population because of its need to establish independence. Considering the AYAs’ developmental stage and their changing lifeworld, a need exists to understand how AYAs, next-of-kin and healthcare professionals may cooperate closely to enact eating support for the AYAs.

Even though the present study was focused on AYAs with cancer receiving HEC, our findings parallel those of studies of next-of-kin to other age groups with cancer. Accordingly, studies of next-of-kin to a child and/or an adult patient with cancer showed the same willingness among next-of-kin to do whatever possible to make the course of treatment as good and painless as at all possible for their loved ones (Fleming et al., 2015; Gibson et al., 2012; Hopkinson, 2018; McCarter et al., 2018; Skolin et al., 2001; Williams & McCarthy, 2015). However, in the interpersonal relationships literature, research has identified that conflict and support often are interrelated (Reblin et al., 2019).

Our findings also highlighted that next-of-kin enacted support by serving as the family’s coordinator, showing care and consideration for everyone in the family and by striving to maintain a normal everyday life even though everything around them seemed chaotic. House et al. (House, 1981; House et al., 1988; Pennant et al., 2019) described social support as a constitution of appraisal, instrumental, informational and emotional functions. Appraisal support involves transmissions of information, which is relevant to self-evaluation. Instrumental support refers to the provision of material of physical assistance such as housing or eating support. Informational support includes providing AYAs with information they may use in coping with personal and environmental problems, and emotional support includes how others’ emotional engagement may counter expressions of distress and facilitate coping (Bell, 2009; Pennant et al., 2019). When the disease emerges and AYAs’ lifeworld is disrupted due to long periods of treatment and devastating side effects, the availability of appraisal, instrumental, informational and emotional support from family and others becomes crucial (Olsen & Harder, 2010; Woodgate, 2006). Olsen and Harder (2009) found that parents and partners often expressed concern of being inadequate in their supporting roles and that a tangible way of supporting AYAs was identified in the next-of-kin’s desire to know and become competent to act. In this process, healthcare professionals play a significant role. One of the cornerstones in the concept of network-focused nursing, developed by Olsen and Harder (2010), is nurses’ communication about the interplay between the AYA, the next-of-kin, and their social network to prevent or resolve potential problems. Supporting the supporters in order to empower them thus becomes an important part of nursing. In this manner, nurses facilitate support of the AYAs through cooperation with and support of the next-of-kin and by encouraging and acknowledging their thoughts and actions in relation to, e.g., eating support (Olsen & Harder, 2010; Riis Olsen & Harder, 2011). This is especially important today as chemotherapy is typically provided as outpatient treatment, thus relying on patient self-care and next-of-kin.

Our findings showed that the next-of-kin are seen as an important resource in providing eating support among AYAs and that health professionals hold the potential to increase the awareness of the complexity of being a next-of-kin and providing eating support for AYAs. Even so, healthcare professionals demonstrate limited awareness of the next-of-kin’s needs in supporting AYAs in the context of eating difficulties.

### Implications for practitioners

This study emphasizes the need to acknowledge next-of-kin as an essential resource in AYAs’ eating support, especially when treatment is provided on an outpatient basis. The findings inform the practices of healthcare professionals as they regularly communicate with the next-of-kin and the AYAs. We highlight the importance of embracing their frustrations and concerns as suggested by Olsen & Harder in their network-focused nursing concept (Olsen & Harder, 2010). Moreover, it is highly relevant for health professionals cooperating with and supporting the next-of-kin in AYAs’ eating difficulties to prepare the next-of-kin for their role as close relative and ensure that they are aware of what to expect when providing eating support during the treatment course. Not only are the next-of-kin in a vulnerable supporting position; they also influence the AYAs’ lifeworld and feel a considerable responsibility for accommodating the eating difficulties faced by the AYAs. They have a need to be well informed and they feel a strong obligation to act the way a parent/next-of-kin should act. Thus, healthcare professionals must involve next-of-kin and their experiences in the early process and respond to their specific information needs.

### Strengths and limitations

Participants were recruited from a single Western European Country, which might have limited the validity of the findings in other contexts as cultural background may influence eating cultures and traditions. A phenomenological study is concerned with gathering in-depth knowledge of the phenomenon under study but not with demographic breadth and ensuring large study samples (Norlyk & Harder, 2010; Van Manen, 2012, 2014) Accordingly, we included 12 next-of-kin with variations in relation to AYAs, their gender and age.

Interviews were conducted as a combination of face-to-face sessions in the homes of the next-of-kin and telephone interviews due to the Covid-19 pandemic. Phenomenological interviews require skills such as active listening, eye contact, nods, and interpretation of non-verbal cues (Van Manen, 2014), which was, of course, difficult to achieve during the telephone interviews. Additionally, it was challenging to get some participants to share their lived experiences as we noticed a preference among next-of-kin for sharing how they thought their loved one experienced eating and not how they experienced being close to them. Conversely, telephone interviews may allow participants to feel more relaxed and able to share sensitive information (Novick, 2008).

A strength of this study is that it was in-depth in nature and had a strong theoretical underpinning that made it possible to understand the meaning of the next-of-kin’s experiences rather than simply describing these experiences. To embed reflexivity in the entire research process we sought to remain aware of the need to keep in check our pre-understandings, assumptions and presuppositions, and we discussed the emerging findings among us until a consensus was reached (Manen van, 1997).

## Conclusions

The present study illuminated the essential meaning of being a next-of-kin who provides eating support to an AYA with cancer. Our findings revealed that utilizing meals as an opportunity to take action towards AYAs’ food intake involved existential feelings of fear of losing their loved ones. Mealtimes essentially evoked an eagerness among next-of-kin to save their AYA from dying from cancer. Eating support was experienced as an unconditional obligation. This prompted the next-of-kin to act; at least, they were able to do something in relation to eating. They experienced walking on a tightrope as they needed to balance their support by insisting but also avoiding pressuring the AYA too much.

The study offers unique, thorough and highly relevant understandings that have implications for clinical practice. It emphasizes that healthcare professionals need to acknowledge next-of-kin as an essential resource in AYA eating support, especially when treatment is provided on an outpatient basis. The findings offer important information relevant to healthcare professionals and especially nurses as they regularly communicate with next-of-kin and AYAs. We highlight the importance of embracing the frustrations and concerns of next-of-kin. In relation to AYAs’ eating difficulties and next-of-kin support, nurses may prepare and guide next-of-kin by preparing them for the challenge of providing eating support at home. Not only are the next-of-kin in a vulnerable supporting position, they also influence the AYAs’ lifeworld and carry responsibility for the wellbeing of the rest of the family aiming to make their everyday life as normal as possible. Thus, healthcare professionals must involve next-of-kin early in the process and respond to their specific information needs in order to avoid their silent suffering due to feelings of frustration and powerlessness. Despite suffering, conflicts and dilemmas, the study clearly indicated that next-of-kin take pride in honouring their responsibilities in supporting AYAs’ eating abilities.
